# Studying and Characterization of Coating of Zein–CaSiO_3_ Composite on Polyetheretherketone Implant Material

**DOI:** 10.1155/2024/1592439

**Published:** 2024-03-16

**Authors:** Sama Abdulrazzaq, Thekra Ismael Hamad

**Affiliations:** Department of Prosthodontics, College of Dentistry, University of Baghdad, Baghdad, Iraq

## Abstract

Material-coated implants are placed in the bone and play an essential role in bone regeneration and rapid healing around implants. Polymeric matrix reinforced with ceramic materials is a promising composite material for coating implants. This study aims to determine the effect of mixing various concentrations of zein with CaSiO_3_ on polyetheretherketone (PEEK) as implant material. The coating was performed using the electrospray method. PEEK disks were used as a control group. The coated disks with different concentrations of zein–CaSiO_3_ (Group 1: 10% wt, 90% wt), (Group 2: 20% wt, 80% wt), and (Group 3%: 30% wt, 70% wt) were the experimental group. Each group was characterized by atomic force microscopy, field emission scanning electron microscope, Fourier-transform infrared spectroscopy, water contact angle, and adhesion strength. The lowest water contact angle was obtained for Group 1: 10% wt and 90% wt were (26.64° and 27.13°, respectively), and increasing amounts of zein in comparison to quantities of CaSiO_3_ resulted in increased adhesion strength of the composite material to the substrate. The current study suggested that the higher amount of zein compared to the amount of CaSiO_3_ mixture coating is achieved by electrospraying, a favorable candidate for coating implants compared to uncoated and coated disks with low concentrations of zein compared to concentrations of CaSiO_3_.

## 1. Introduction

In recent years, dental implants have become one of the leading treatments to replace missing teeth. Compared to conventional methods such as dentures, partial dentures, and complete dentures, dentures offer benefits such as esthetics, comfort, and keeping of the natural teeth and bone living tissue [1, 2].

Demand for dental implants has fueled rapid market expansion and the development of new surgical and prosthodontic techniques; implant dentistry continues to evolve and expand [3].

Polyetheretherketone (PEEK) is a thermoplastic polymer with high performance that is capable of replacing metallic implant components in orthopedics and traumatology. Such findings suggested that PEEK be a substitute for titanium as dental endosseous implant material [4].

An aromatic backbone with ketone and ether functional groups between the aryl rings makes up PEEK. It is in the polyarylethrketone family. It is a high-performance engineering plastic with many excellent properties, including chemical stability, heat and machinability, frictional resistance, good biocompatibility, an elastic modulus close to human bone, and good X-ray penetration [5]. PEEK's excellent mechanical properties, minimal immunotoxicity, and overall good processing performance have made it an alternative to metal implants and orthopedics [6]. PEEK can overcome some of the limitations of metal implants, such as stress shielding and metal allergy [7].

As an implanted biomaterial, PEEK has a modulus of elasticity of unmodified PEEK of 3–4 GPa, a close match to that of cortical bone. On the other hand, PEEK is bioinert and has poor cellular adhesion properties due to its hydrophobicity [8, 9].

The bioinertness of pristine PEEK hinders integration with native bone tissues *in vivo*, so improvement is needed. To reinforce the osseointegration ability of PEEK prostheses, surface modification strategies such as electron beam evaporation, plasma treatment, and wet chemistry have been suggested [10].

Successful implant surface treatment has promoted bone growth and attachment, a process called osseointegration [11]. The response of osteoblasts (bone-forming cells) to the implanted material depends on the material's surface properties regarding topography and surface chemistry [2, 12].

One of the most critical factors in determining the clinical success of implantation is osseointegration, defined as “a direct relation of structure and function between ordered living bone and the surface of a supporting implant” [13].

Advantageously, plant proteins are renewable and inexpensive, making them suitable for biomedical research. These properties make plant proteins more credible than animal proteins [14]. Zein is an insoluble prolamin protein derived from corn. It became accredited via the FDA as GRAS (generally recognized as safe) in 1985 [15].

Calcium silicate (CaSiO_3_) has been proposed as a potential bone regeneration material due to its demonstrated bioactivity, excellent biocompatibility, high mechanical properties, and degradability [16–18].

Calcium silicate (CaSiO_3_) nanoparticles enhance the implanted prosthesis's bone formation. These bioactive ceramics can enhance new bone tissue formation by creating a tight bond between the implant and the host bone after implantation [19].

The apatite formation rate on the CaSiO_3_ surface is even higher than other bioglasses and glass ceramics in simulated body fluids [20]. In this study, different percentages of zein/CaSiO_3_ composite will be formed on the surface of dental PEEK implants by electrospraying coating. Electrospraying is one of the valuable techniques for obtaining a uniform coating; an electric potential is applied to the coating material. At this point, opposing forces come into play: the surface tension and viscoelastic forces of the coating material, which tend to maintain the hemispherical shape of the coating, and the electrical field-induced charge [21].

The electrospray zein/calcium silicate composite's characteristics as a coating material for PEEK implants that will affect the healing quality around implants have not been studied. However, calcium silicate ceramics have certain shortcomings for application as coating materials. First, they have a high dissolution rate, and the excessive release of Ca and Si ions can inhibit cell proliferation. Second, the mechanical strength of calcium silicate is below the range required for cortical and cancellous bone regeneration, which reduces the ability of the material to maintain mechanical stability under physiological loading. In addition, calcium silicate's osteoinductivity and bone ingrowth properties remain inadequate. Owing to these limitations, researchers have endeavored to improve calcium silicate's mechanical and osteogenic properties by adding various materials [22]. So, the novelty of this work is to evaluate if the addition of protein material to calcium silicate affects the performance of calcium silicate as a coating material; therefore, this study aims to investigate some properties of various concentrations of zein and calcium silicate composite coating (with different concentrations of 10% : 90%, 20% : 80%, and 30% : 70% of zein : calcium silicate) on PEEK dental implants.

## 2. Materials and Methods

### 2.1. Preparation and Cleaning of Medical-Grade PEEK Implant Material Disks

Disc-shaped samples will be prepared from medical-grade PEEK material. For the zein/CaSiO_3_ coating, use disk samples that are 2 mm thick and 10 mm in diameter.

#### 2.1.1. Surface Modification of PEEK

Disc samples were etched in H_2_SO_4_ (98%, M.W. 98.08) for 60 s, then washed with water and ethanol [23].

#### 2.1.2. Preparation of Zein–CaSiO_3_ Composite Solutions

Zein–CaSiO_3_ solutions with three different zein–CaSiO_3_ concentrations (Group 1: 10% by weight, 90% by weight), (Group 2: 20% by weight, 80% by weight), and (Group 3: 30% by weight, 70% by weight), respectively, were prepared by dissolving zein protein powder in ethanol on a magnetic stirrer for 30 min, then adding CaSiO_3_ and dispersing the suspension in probe sonication for 10 min to ensure complete dispersion of ceramic material.

#### 2.1.3. Electrospraying Process

PEEK disks were coated using an electrospraying process. The solution was pumped by a syringe pump (USA, Era 300N, high-tension power supply, 30 kV, China) at a flow rate of 1.5 ml/hr through a metallic needle (needle gauge 21 microns). The capillary-collected distance was 5 cm, with a 9 kV voltage source. The electrospraying process was done at first on an aluminum foil to adjust the electrospraying parameters; after that, the coating was done on a PEEK substrate.

### 2.2. Characterization of Coating Composites

#### 2.2.1. Atomic Force Microscopy (AFM) Test

AFM is one of the most often used and practical techniques for detecting surface morphology, roughness, and topography. AFM consists of a cantilever with a sharp, highly sensitive probe tip that contacts the material surface.

#### 2.2.2. Scanning Electron Microscope (SEM)

SEM was used to examine the surface shape of PEEK disks with three concentrations of zein–CaSiO_3_ added, along with control disks and disks with only the surface treated.

#### 2.2.3. Fourier Transmission Infrared Spectroscopy (FTIR) Analysis of Coating Material

Chemical bonds in molecules are identified by taking an infrared absorption spectrum using FTIR (8400S, Shimadzu, Japan). The spectra create a sample profile that is considered a unique molecular fingerprint that can be used to scan and screen samples for many different components.

### 2.3. Wettability Test

The water contact angles of the PEEK disks with varied zein–CaSiO_3_ coating concentrations were measured and compared to control disks to identify which disks had improved hydrophobicity. The water contact angle image was taken using (Si-Plasma Cam 110, Creating Nano Technologies, Taiwan) after a drop of distilled water was applied using a small syringe to the target surface. An image could be taken once a drop of liquid had been placed on the desired surface for 30 s at room temperature.

### 2.4. Adhesion Test

All the coated specimens were subjected to adhesion (pull-out) testing to investigate the adhesion bond between the composite and PEEK disks.

## 3. Results

**Figure 1:**
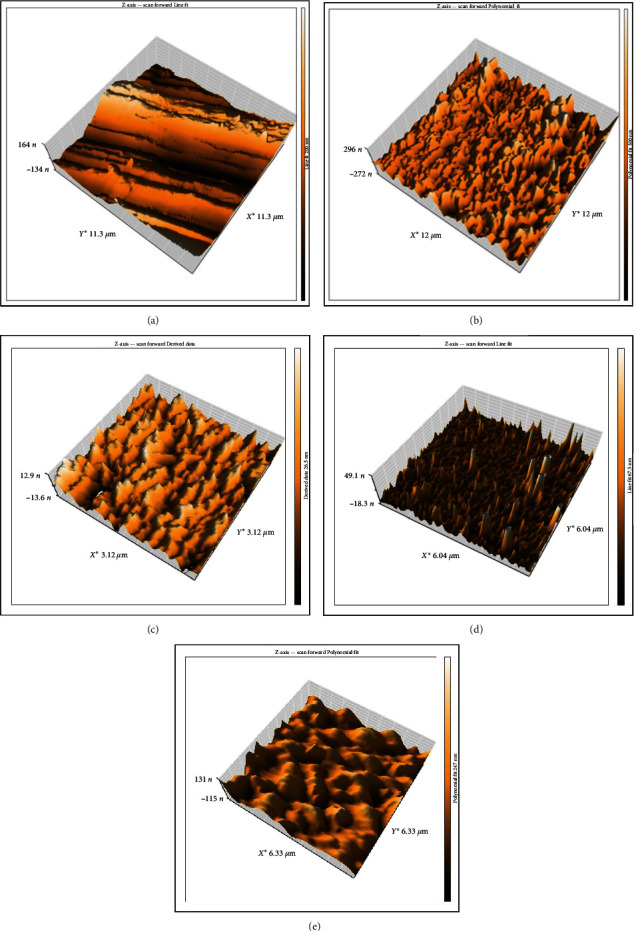
AFM analysis for (a) control PEEK, (b) the modified surface of PEEK, (c) Group 1 (10% zein–90% CaSiO_3_), (d) Group 2 (20% zein–80% CaSiO_3_), and (e) Group 3 (30% zein–70% CaSiO_3_).  ^*∗*^denotes the height measured as a function of X and Y position.

Surface modification for PEEK with H_2_SO_4_ acid increased surface roughness for PEEK about five times and was analyzed by AFM, as shown in Figure 1(b). The electrospraying processing variables are optimized, as in Table 1.

For all PEEK specimens (control, modified surface with H_2_SO_4_, and coated), surface roughness analysis was evaluated by AFM (Figure 1) to detect the number of morphological changes on the surface, which is one of the essential aspects of implant success with coating. Surface roughness for control, modified texture with H_2_SO_4_, and coated specimens are listed in Table 2.

**Figure 2:**
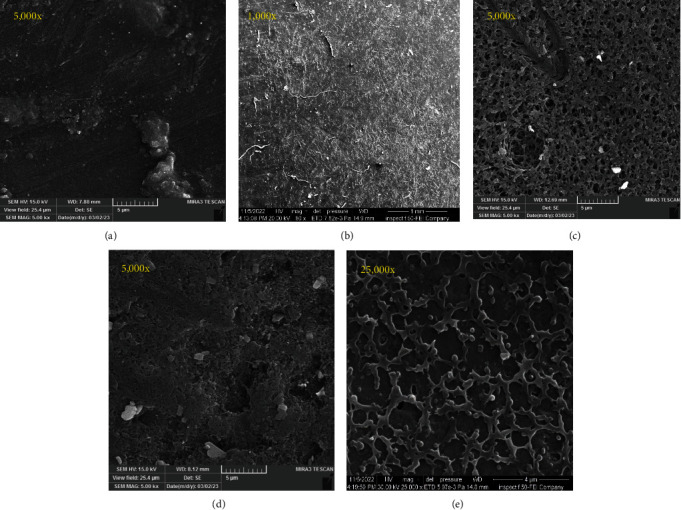
SEM analysis (a) control, (b) modified surface with H_2_SO_4_, (c) Group 1 (10% zein–90% CaSiO_3_), (d) Group 2 (20% zein–80% CaSiO_3_), (e) Group 3 (30% zein–70% CaSiO_3_).

Surface morphology is recognized to significantly impact cell behavior, with pore size, pore connectivity, and surface properties having the most influence on interactions between cells and surfaces of implants, including cell adhesion, migration, and proliferation. At high-magnification images of the composite-based samples, they revealed no cluster of the protein particles with bioceramic material, indicating that the two materials were mixed adequately before electrospraying, according to SEM observations of the controlled, modified surface and coated PEEK with varied percentages, as shown in Figure 2.

**Figure 3:**
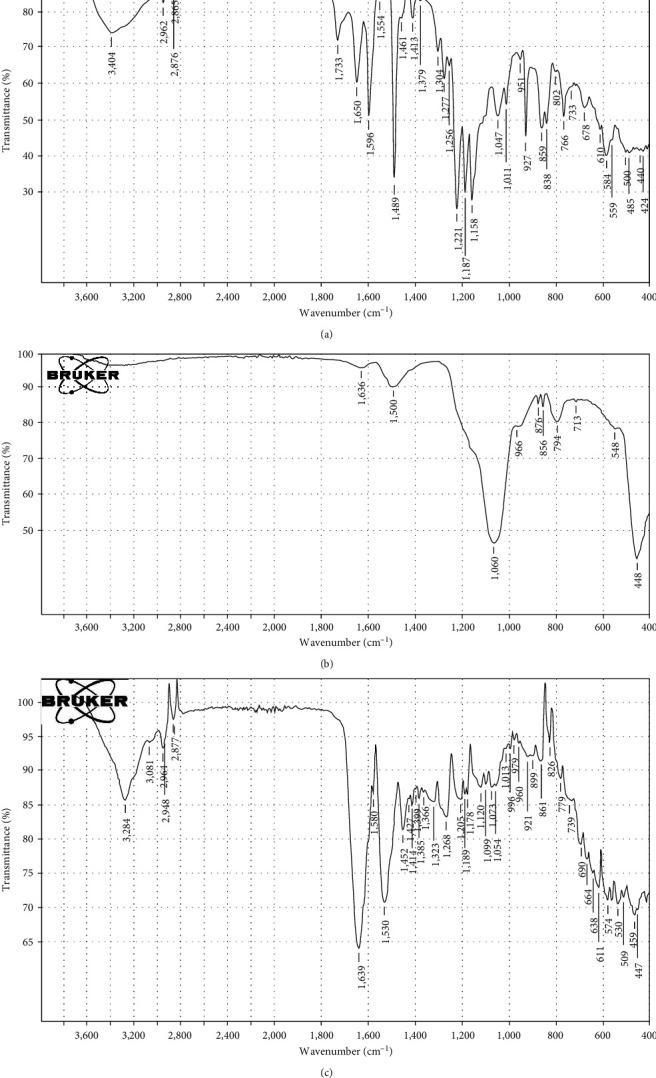
FTIR analysis for (a) mixture of zein–CaSiO_3_, (b) calcium silicate, and (c) zein.

The mixed zein–CaSiO_3_ powder was subjected to FTIR analysis to establish the inclusion of zein–CaSiO_3_ inorganic phases, as shown in Figure 3. FTIR of calcium silicate showed Si─O─Si bending vibrations give absorption bands also in the range of 548 cm^−1^. The band at 856 cm^−1^ relates to the symmetric stretching of Si─O─Si. About 1,060 and 966 cm^−1^ came from asymmetric stretching of Si─O─Si, Si─O─Ca [24, 25]

The three characteristic peaks of zein at 1,639, 1,530, and 1,366 cm^−1^ correspond to amide I (C = O), amide II (N─H bend), and amide III (C─N), respectively. The carboxyl groups are present in the spectrum at about 2,948 cm^−1^ [26]. In the mixture of calcium silicate/zein, the band of Si─O─Si bending at 559 cm^−1^, at 859 the symmetric stretch of Si─O─Si, 951 show the band of Si─O─Ca, 1,047 the band of Si─O─Si. In 1,554, 1,650, 1,379, and 2,962 cm^−1^, the bands of amide I, II, III, and carboxyl group, respectively.

### 3.1. Wettability Test

**Figure 4:**
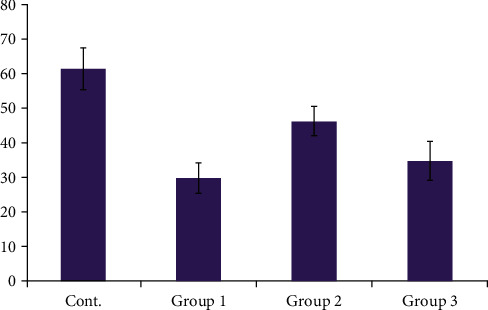
Comparison of water contact angle of all studied groups.

Water contact angle images were taken for all study groups, as shown in Figure 4. The mean of water contact angle for control PEEK specimens was (61.473°), while the PEEK coated with (10% zein—90% CaSiO_3_; Group 1) showed the lowest water contact angle (29.839°), with statistical analysis shown in Tables 3–5.

### 3.2. Adhesion Test

**Figure 5:**
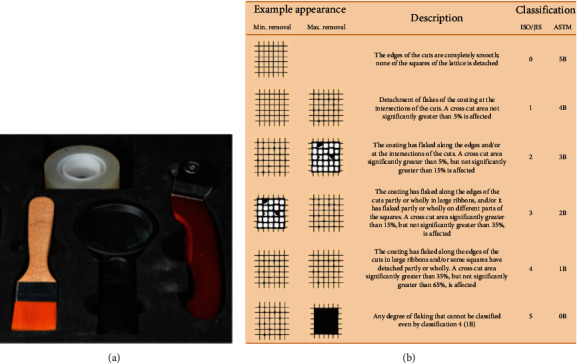
(a) Adhesion cross-hatch test kit and (b) cross-cut guidance.

A cross-cut, scratch adhesion test determines a coating's resistance to delamination from a substrate by using a tool to cut a rectangular grid pattern into the coating and penetrate the substrate. This method performs a quick pass/fail test. When testing multilayer structures, the resistance of different layers to separation from each other can be determined. This method is standardized by ASTM D 3359 [27]. A six-step classification is given in cross-cut test guidance (Figure 5). The first three steps are satisfactory for general purposes and will be used when a pass/fail assessment is required. Exceptional circumstances might arise where the complete six-step classification will be necessary.

The findings demonstrated that the strength of adhesion increases with increasing amounts of zein protein in comparison to quantities of CaSiO_3_, as shown in Tables 6–8.

## 4. Discussion

PEEK is chemically inert due to its aromatic chain structure with a combination of ketone and ether functionalities between the aryl rings. However, sulfonation of PEEK in concentrated sulfuric acid successfully introduces a 3D porous nanostructured mesh and SO_3_H groups onto its surface. Sulfonated PEEK improves all osteoblast capabilities, including preliminary molecular adhesion, molecular viability, proliferation, differentiation, bone regeneration, and apatite formation [28].

It was observed that surface-modified PEEK stimulated the proliferation of osteoblasts, mRNA synthesis, and collagen I turnover comparable to smooth and rough titanium. Modified PEEK and titanium implants stimulated cellular differentiation and proliferation in comparable magnitude. In a series of studies by Olivares-Navarrete et al. [29], results indicate that although PEEK stimulates cellular proliferation, the cells proliferating on PEEK are less osteoconductive than those on titanium. In vivo studies suggest that PEEK favors lesser production of pro-osteoblast proteins than titanium, further strengthening the notion that PEEK is less osteoconductive than titanium.

When Koch et al. [30] compared the osseointegration of titanium, coated and uncoated zirconia, and PEEK implants, significantly less bone-implant contact was observed around PEEK implants; similar observations were made by Webster et al. [31] who also observed a significantly higher susceptibility of bacterial growth on PEEK implant surfaces, which may be an additional factor playing a role against diminished osseointegration of PEEK implants. Results from Nakahara et al. [32] suggest that the limited osteoconductive properties of PEEK may be overcome by coating it with bioactive materials. A major cause of concern for uncoated PEEK is its low wettability and, hence, high hydrophobicity, which may prevent initial cellular adhesion. Several methods have been proposed to improve the bioactivity of PEEK, including coating PEEK with synthetic osteoconductive hydroxyl apatite, increasing its surface roughness and chemical modifications, and incorporating bioactive particles [33, 34].

Surface chemistry, nanotopography, porosity, and roughness are key characteristics affecting optimal osteoconductivity. A sulfonation treatment was developed to facilitate PEEK's hydrophilicity and surface morphology for bone implants. For reference, the hydroquinone ring alongside the ether bridge should be sulfonated. By treating the surface, charged sulfonate groups were added to the aromatic PEEK ring, and PEEK was changed into sulfonated PEEK (SPEEK-H) [35].

Micro- and nanoscale topography and appropriate surface roughness have been manifested to affect cell behavior and the formation of bone. Proper surface roughness promotes the involvement of extracellular matrix proteins, whichever is crucial for the initial adhesion of the cell [36]. The roughness of the coating decreased as the concentration of ceramic material increased. This behavior can be explained by the fact that the particles in the coating became more densely packed with an increase in the concentration of ceramic material, resulting in a smoother coating surface [37].

The deposition of organic and inorganic composites onto implant materials has been explored to modify the surface of implants. These composite coatings can prompt osseointegration with host tissue and evolve bulk and implant surface properties [38].

The electrospraying technique gave the coated disks a rough surface of about 81.59 nm, much more intimidating than the sulfonated-treated disks and the control group. The electrospray deposition produced uniform coverage of the PEEK substrate with the CaSiO_3_/zein composite. Coatings were analyzed under scanning microscopy, and a homogeneous coating is apparent without cracks for all coating groups, with a meshwork of incompletely merged zein globules observed that agreed with the result of Clavijo et al. [38].

The water contact angle showed a high affinity for water; ceramic material can function as a surfactant and reduce the surface tension of the contacting liquid, which may be the cause of the static contact angle's decline. Another explanation for the increase in wettability may be the surface roughness brought on by including nanoparticles. Roughness relates to contact angle, so if less than 90°, it increases hydrophilic with an increase in nanofiller. Still, if the contact angle is more than 90°, this shows a more hydrophobic property [39]. The 20% and 30% zein coatings exhibited lower hydrophilicity than those with 10% zein. Thus, zein/calcium silicate coatings improved the surface properties of PEEK. Zein is amphiphilic in character. Its hydrophobicity is due to the presence of amino acids (such as proline, leucine, isoleucine, and alanine), whereas its hydrophilicity is due to glutamine. The calcium silicate incorporation enhances the hydrophilic nature of zein, and thus, the combination of both leads to the overall hydrophilic nature of the coating. Moreover, it may also be possible that the glutamine chains were present at the top of the coatings, which may have led to the hydrophilicity of the coatings [40]. Compared to the untreated substrates, the coatings were rougher and more densely packed. It has been demonstrated that the substrates' surface preparation significantly affects the coating's adherence [38]. The surface preparation of the underlying substrates has a significant impact on the zein coatings' ability to adhere to them. It was discovered that surface characteristics and chemistry had a bigger impact on coating adherence than substrate surface roughness [41]. For cross-cut adhesion tests, increasing the amount of zein within the coatings could improve the mechanical strength of the coatings. Batool et al. [42] demonstrated that a polymer matrix (e.g., zein) can act as a binder to hold another material on the surface and thus increase the mechanical stability of coatings.

This study was limited to studying the mechanical properties of different concentrations of zein with a calcium silicate composite coating on PEEK implant material. This study could not clarify the effect of humidity, temperature, flow rate, and needle diameter on the electrospraying process. Future research could investigate the performance of zein/calcium silicate composite as a coating material on osseointegration and compatibility with animals. The clinical significance of coating material is that it has a notable effect on the quantity and quality of osseointegration around implants.

## 5. Conclusion

The present study evaluates the effect of zein reinforced with different concentrations of CaSiO_3_ as a coating material on PEEK. Significantly, including a higher concentration of zein in bioceramic material improved the coating material's hydrophilicity performance and increased the adhesion strength of the coating material to the substrate.

## Figures and Tables

**Table 1:** The electrospraying processing variables.

Conc. of zein–CaSiO_3_ (W/V %)	Flow rate (ml/hr)	Voltage (kV)	Distance (cm)
Zein–CaSiO_3_	1.25	4.5	5
	1.5	5	5
	1.5	8	5
	1.5	9	5

**Table 2:** Amount of roughness (nm) according to AFM analysis.

Groups	Roughness (Ra) (nm)
Control	3.248
Modification with H_2_SO_4_	8.726
Group 1	33.56
Group 2	35.90
Group 3	81.59

**Table 3:** Statistical analysis of wettability test for all studying groups.

Group	Mean	*N*	Std. deviation
Cont.	61.473	10	5.969
Group 1	29.839	10	4.391
Group 2	46.323	10	4.207
Group 3	34.868	10	5.615

**Table 4:** ANOVA analysis of wettability test for all studying groups.

	Sum of squares	df	Mean square	*F*	Sig.
Between groups	5,915.579	3	1,971.860	75.718	0.000 ^*∗*^
Within groups	937.513	36	26.042		
Total	6,853.091	39			

^*∗*^The mean difference is significant at the 0.05 level.

**Table 5:** LSD analysis of wettability test for all groups.

Group	Groups	Mean difference	Std. error	Sig.
Cont.	Group 1	31.63340 ^*∗*^	2.28219	0.000
	Group 2	15.15000 ^*∗*^	2.28219	0.000
	Group 3	26.60510 ^*∗*^	2.28219	0.000

Group 1	Group 2	−16.48340 ^*∗*^	2.28219	0.000
	Group 3	−5.02830 ^*∗*^	2.28219	0.034

Group 2	Group 3	11.45510 ^*∗*^	2.28219	0.000

^*∗*^The mean difference is significant at the 0.05 level.

**Table 6:** Statistical analysis of adhesion test for all studying groups.

Groups	Mean	*N*	Std. deviation
Group 1	1.3000	10	0.48305
Group 2	1.5000	10	0.52705
Group 3	0.4000	10	0.51640

**Table 7:** ANOVA analysis of adhesion test for all studying groups.

	Sum of squares	df	Mean square	*F*	Sig.
Between groups	6.867	2	3.433	13.243	0.000 ^*∗*^
Within groups	7.000	27	0.259		
Total	13.867	29			

^*∗*^The mean difference is significant at the 0.05 level.

**Table 8:** LSD analysis of adhesion test for all studying groups.

Group	Groups	Mean difference	Std. error	Sig.
Group 1	Group 2	−0.20000	0.22771	0.388
	Group 3	0.90000	0.22771	0.001 ^*∗*^

Group 2	Group 3	1.10000	0.22771	0.000 ^*∗*^

^*∗*^The mean difference is significant at the 0.05 level.

## Data Availability

All data are included in the article.
